# A hemodynamic and biomarker study of pulmonary hypertension associated with heart failure: DISCOVER PH-HF

**DOI:** 10.2459/JCM.0000000000001872

**Published:** 2026-05-07

**Authors:** Roberto Manni, Henri Xhakupi, Suela Vani, Soulef Bouakkaz, Monica Marchese, Paola Visconti, Fabio Pescetelli, Matteo Toma, Gaetano Maria De Ferrari, Marianna Adamo, Pietro Ameri, Italo Porto

**Affiliations:** a Department of Internal Medicine; b Department of Experimental Medicine, University of Genova; c Centre of Biological Resources; d Cardiovascular Disease Unit, IRCCS Azienda Ospedaliera Metropolitana, Genova; e Cardiology, Department of Medical Sciences, University of Torino and AOU Città della Salute e della Scienza Torino; f Department of Medical and Surgical Specialties, Institute of Cardiology, ASST Spedali Civili, Radiological Sciences, and Public Health, University of Brescia, Brescia; g Translational Cardiovascular Unit, IRCCS Azienda Ospedaliera Metropolitana, Genova, Italy

**Keywords:** hemodynamics, mechanism, pathobiology, translational

## Abstract

**Aims:**

The pathogenesis of pulmonary hypertension associated with heart failure (PH-HF) is partially understood. To advance knowledge in this regard, the investigator-initiated, public-funded, prospective, translational stuDy to dISseCt remOdeling of capillaries and Veins in pulmonary hypErtension associated with heaRt failure (DISCOVER PH-HF) will assess biomarkers of PH-HF in pulmonary capillary and venous (PCV) and peripheral venous blood taken during right heart catheterization (RHC). This report presents the design of DISCOVER PH-HF and the baseline characteristics of the included patients.

**Methods:**

DISCOVER PH-HF enrolled heart failure patients scheduled for transcatheter edge-to-edge repair (TEER) of severe mitral regurgitation. PCV and peripheral venous whole blood was collected during RHC within 48 h before mitral regurgitation (MR)-TEER, and processed into plasma/serum aliquots and peripheral blood mononuclear cell (PBMC) pellets. Then, the samples were centralized to a core biobank. Follow-up entails visits at 90 ± 10 and 180 ± 10 days and – optionally – a second RHC with collection of PCV and peripheral venous blood within 30 days from the second follow-up evaluation. A proteomic analysis of part of the PCV and peripheral plasma is already planned.

**Results:**

Seven patients without pulmonary hypertension at RHC, 12 patients with isolated postcapillary pulmonary hypertension, and 18 patients with combined postcapillary and precapillary pulmonary hypertension were recruited, leading to a total of 148 PCV and 148 peripheral plasma aliquots, 111 PCV and 111 peripheral serum aliquots, and 37 PBMC pellets.

**Conclusion:**

DISCOVER PH-HF has generated a limited-size, but unique biobank of RHC-derived PCV and peripheral venous blood samples and PBMCs, linked to clinical and hemodynamic phenotyping, which will allow the exploration of biomarkers of PH-HF and its subtypes.

## Graphical abstract

**Figure:**
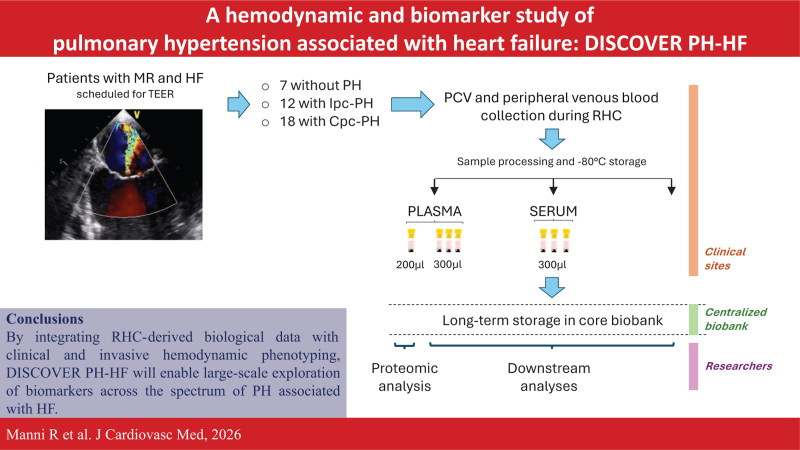

## Introduction

Pulmonary hypertension(PH) is a common complication and compounds the prognosis of left heart failure, especially with concomitant mitral regurgitation (MR).^1^

PH associated with heart failure (PH-HF) is initially caused by the retrograde transmission of high filling pressures from the left cardiac chambers to the pulmonary circulation. In some individuals, the backward pressure elevation, or isolated postcapillary pulmonary hypertension (Ipc-PH), is accompanied by functional and structural remodeling of small pulmonary vessels, which generates resistance to blood flow and further raises pulmonary artery pressure (PAP).^2^ The term combined postcapillary and precapillary pulmonary hypertension (Cpc-PH) is used to define this stage of disease, based on the demonstration of the remodeling of pulmonary arterioles similar to that occurring in pulmonary arterial hypertension.^3^ However, more recent investigations have revealed that pulmonary capillaries and venules (PCV) also undergo pathological changes in PH-HF.^4^

The distinction between Ipc-PH and Cpc-PH relies on right heart catheterization (RHC).^5^ According to the current European Society of Cardiology/European Respiratory Society guidelines on pulmonary hypertension, both conditions are characterized by mean PAP greater than 20 mmHg and pulmonary artery wedge pressure (PAWP) greater than 15 mmHg, but pulmonary vascular resistance (PVR) is 2 WU or below in Ipc-PH and above 2 WU in Cpc-PH.^5^ The adoption of this cutoff has been prompted by the observation that, in large datasets, outcomes were worse in individuals with PVR above than below 2 WU.^6^ It remains unclear whether the haemodynamic threshold of PVR above 2 WU reflects a true biological transition in pulmonary vascular disease, rather than being a purely prognostic marker. In particular, it has not been established whether this cutoff corresponds to distinct patterns of pulmonary microvascular remodeling, including involvement of PCV. Integration of invasive hemodynamics with compartment-specific biological investigations may provide answers to these questions.

Factors released by PCV are enriched in blood drawn through the wedged Swan-Ganz catheter during RHC, in which they can be measured. Moreover, assessment of these factors in simultaneously collected peripheral venous blood allows quantification of their relative abundance in the PCV compartment.

By exploiting RHC performed before the transcatheter edge-to-edge repair (TEER) of MR, the translational stuDy to dISseCt remOdeling of capillaries and Veins in pulmonary hypErtension associated with heaRt failure (DISCOVER PH-HF) was conceived to set up a unique biobank of RHC-derived PCV and peripheral venous blood samples for the discovery of biomarkers of the biological underpinnings of PH-HF, including the subtypes Ipc-PH and Cpc-PH.

In this article, we describe the study design and the baseline characteristics of the included patients.

## Methods

### Study design

DISCOVER PH-HF is an investigator-initiated, public-funded, prospective, translational study carried out at three large-volume centers for MR-TEER (Ospedale Policlinico San Martino/IRCCS Azienda Ospedaliera Metropolitana, Genova; A.O.U. Città della Salute e della Scienza di Torino, Torino; A.S.S.T. degli Spedali Civili, Brescia), in which RHC is routinely done shortly before the percutaneous intervention.

The study was designed to systematically collect PCV and peripheral venous blood during RHC before the TEER of MR with heart failure, for subsequent proteomic analysis and other targeted and untargeted assays aiming to pinpoint the biomarkers of PH-HF, Ipc-PH, and Cpc-PH. Peripheral blood mononuclear cells (PBMCs) were also collected for later DNA and RNA extraction and sequencing.

Patients were then asked to attend an ambulatory visit with transthoracic echocardiography 90 ± 10 and 180 ± 10 days after the initial RHC. Furthermore, another RHC with collection of PCV and peripheral venous blood was proposed within 30 days from the second follow-up visit (200–220 days after the baseline RHC), if no more than mild residual mitral regurgitation was present. While the baseline RHC was done as per the standard of care at the DISCOVER PH-HF centers, the follow-up one was exclusively performed for research purposes.

Thereafter, patients were reached by telephone for an update on their status every 6 months until the end of the study.

DISCOVER PH-HF was approved by the local Ethics Committees CET Liguria (N. 268/2024), CET Lombardia 6 (N. 2025-3.11/64), and CET Interaziendale A.O.U. Città della Salute e della Scienza di Torino (N. 00083/2025). The study participants signed written informed consent to the study and another to biobanking, in full compliance with local data protection regulations and GDPR requirements.

### Patients

Eligible patients had MR with heart failure and were listed for RHC before TEER. Moreover, the following were exclusion criteria: in need of uninterrupted nitrate or sodium nitroprusside infusion to maintain clinical stability; previous or concomitant PH other than PH-HF; known intracardiac shunt; current therapy with pulmonary vasodilators; ongoing acute or chronic inflammatory condition, as inferred by clinical examination and/or C-reactive protein concentration greater than 10 mg/l within 3 days before enrolment; and participation in any other interventional study.

A baseline RHC was done ≤48 h before the TEER, at rest and under fasting conditions. Oxygen saturation (SpO_2_) was determined at multiple levels in superior and inferior vena cava, right cardiac chambers, and pulmonary artery (PA) to confirm the absence of intracardiac or intrapulmonary shunt. Systemic blood pressure, heart rate, systolic PAP, diastolic PAP, mPAP, PAWP, cardiac output (estimated by thermodilution or, in the case of severe tricuspid regurgitation, the indirect Fick method), and right atrial pressure were directly measured. Secondary parameters, such as stroke volume, cardiac index, and PVR, were calculated from primary, directly assessed parameters.

At the end of RHC, 2 ml of PCV blood were drawn through the tip of the Swan-Ganz catheter in the wedged position, as verified by pressure waveforms and SpO_2_, and 2 ml of peripheral venous blood were retrieved by venipuncture.

Any infusion of nitrate or sodium nitroprusside for treatment of heart failure was stopped 30’ before starting RHC to avoid potential effects of these drugs on pulmonary endothelial cells and their secretome.

### Blood sample processing

**Fig. 1:**
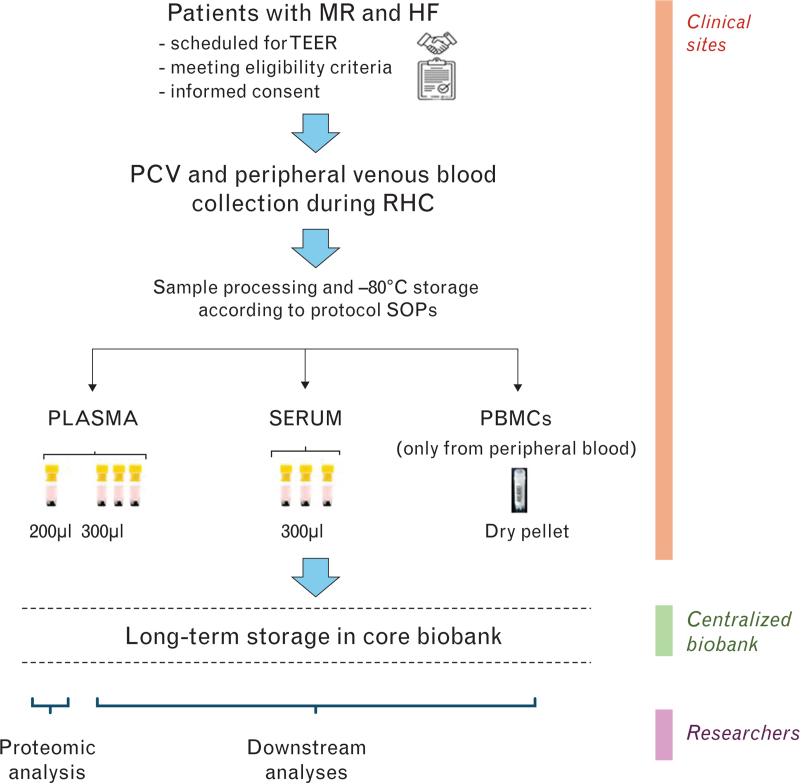
Workflow of DISCOVER PH-HF. Upon signing the written informed consent, eligible patients with mitral regurgitation (MR) and heart failure (HF) underwent right heart catheterization (RHC) with collection of pulmonary capillary and venules (PCV) and peripheral venous blood. At each study site, plasma and serum aliquots were obtained from whole blood from both the pulmonary and the systemic circulation according to preestablished standard operating procedures (SOPs). Moreover, peripheral blood mononuclear cells (PBMCs) were isolated from peripheral blood. These biological samples were initially stored at the sites where they were collected. Then, they were centralized to the core DISCOVER PH-HF biobank for long-term storage until use, either as already planned (proteomic analysis) or to be still defined by the study scientific committee, autonomously or within collaborations with other researchers.

PCV and peripheral venous whole blood was processed by centrifugation at 2000*g* for 10 min at room temperature within 30 min from collection, to obtain EDTA plasma and serum. Plasma was split into one 200-μl aliquot for Olink analysis and three 300-μl aliquots for downstream analyses, while serum was only distributed into three 300-μl aliquots. Plasma and serum cryovials were then kept at −80 °C. PBMCs were isolated from peripheral venous blood, pelleted, dried, and stored at −80 °C (Fig. 1).

### Biomarker studies

DISCOVER PH-HF entails two different strategies to explore biomarkers of PH-HF and its subtypes.

Part of the collected blood will be used for proteomic analysis with the Olink proximity extension assay. Briefly, this technology consists of two steps: recognition of target proteins by two matched antibodies labelled with unique DNA oligonucleotides; DNA polymerase-dependent extension of the double-stranded DNA that forms through hybridization of the antibody oligonucleotides. The resulting DNA amplicons are read on a sequencing platform, with the output signal being proportional to the initial amount of the target proteins. More than 5000 unique proteins, broadly related to cardiovascular physiology and pathology, metabolism, inflammation, differentiation, survival, and proliferation, will be screened. Differentially expressed proteins will be identified between patient subgroups (no-PH, Ipc-PH and Cpc-PH) by applying log fold-change, *P* value, and adjusted *P* value thresholds of ≥2, <0.005, and <0.1, respectively. If these cutoffs prove to be too restrictive, the lower one will be used. Both batch effects and clinically relevant covariates will be accounted for. The pathways related to the differentially expressed proteins will be evaluated by interrogating resources such as Gene Ontology (GO) and Kyoto Encyclopedia of Genes and Genomes (KEGG).

The remaining plasma and serum aliquots will be stored at the Centre of Biological Resources of Ospedale Policlinico San Martino/IRCCS Azienda Ospedaliera Metropolitana, which serves as the core biobank for DISCOVER PH-HF, and will be made available for future investigations. The Centre of Biological Resources is certified (ISO9000-2015, OECI), recognized by local institutions (DGR n. 34 Regione Liguria, 22/01/2010) and participates in the Biobanking and BioMolecular Resources Research Infrastructure of Italy (BBMRI, https://www.bbmri.it/; part of the European research infrastructure for biobanking BBMRI-ERIC).

The collected samples from subjects enrolled at Ospedale Policlinico San Martino/IRCCS Azienda Ospedaliera Metropolitana were directly handled by the staff of the Centre of Biological Resources. The samples collected at the other two DISCOVER PH-HF centers were processed and initially stored at those sites and shipped to the core biobank at enrolment completion (Fig. 1).

For each recruited subject, the polypropylene cryovials containing plasma, serum, and PBMCs were linked to a pseudonym code, recorded in the core biobank informatic system, and labelled with a 2D barcode, which can be scanned for tracking and inventory.

### Sample size

As this study is exploratory, the sample size could not be estimated *a priori*. The minimum target was at least 10 patients with Ipc-PH and at least 10 patients with Cpc-PH.

### Statistical analysis

All statistical analyses are descriptive, and no causal inference is implied.

The clinical characteristics and pulmonary hemodynamics at baseline are presented as number (proportion of total), mean ± standard deviation, or median (interquartile range) and were compared among patient groups by chi-square test, Friedman's analysis of variance (ANOVA), or Kruskal–Wallis test with Bonferroni correction, respectively. A *P* value less than 0.05 was considered statistically significant.

## Results

**Fig. 2:**
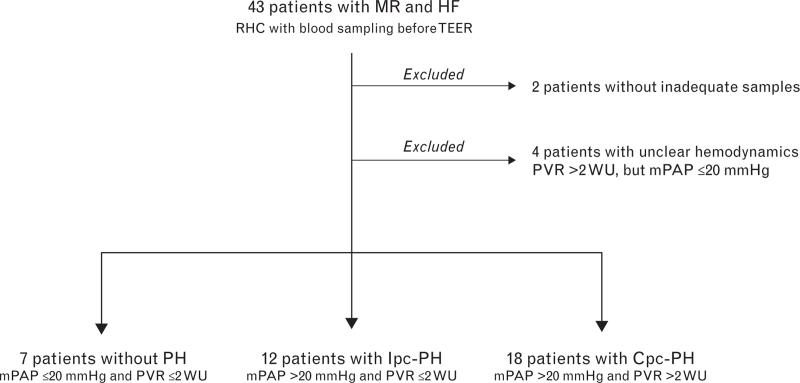
CONSORT diagram depicting the enrolment in DISCOVER PH-HF. Cpc-PH, combined postcapillary and precapillary pulmonary hypertension; HF, heart failure; Ipc-PH, isolated postcapillary pulmonary hypertension; mPAP, mean pulmonary arterial pressure; MR, mitral regurgitation; PH, pulmonary hypertension; PVR, pulmonary vascular resistance; TEER, transcatheter edge-to-edge repair; RHC, right heart catheterization.

Forty-three patients with mitral regurgitation and heart failure were enrolled in DISCOVER PH-HF. Two were excluded from the study after RHC because the quality of the collected PCV blood samples was inadequate to follow the established processing procedures. Another four had an ambiguous hemodynamic profile with mPAP 20 mmHg or less, but PVR 2 WU or below. Of the remaining 37 patients, 7 did not have pulmonary hypertension at RHC (i.e. mPAP was ≤20 mmHg and PVR was ≤2 WU), 12 had Ipc-PH, and 18 had Cpc-PH (Fig. 2).

Their characteristics are presented in Table 1 and pulmonary hemodynamics are summarized in Table 2. Concentrations of N-terminal pro-brain natriuretic peptide were progressively higher, and the use of diuretics was progressively more frequent across the no-PH, Ipc-PH, and Cpc-PH groups. Furthermore, hemoglobin levels were lower in patients with PH-HF than without PH-HF. By definition, mPAP and PVR increased from patients without pulmonary hypertension to those with Ipc-PH to those with Cpc-PH, and PAWP was greater in subjects with than without Ipc-PH and Cpc-PH. There was also a trend for higher right atrial pressure in the former than in the latter. Cardiac index was lower in patients with Cpc-PH than in the others.

**Table 1:** Baseline characteristics of the patients without pulmonary hypertension, with isolated postcapillary pulmonary hypertension, and with combined postcapillary and precapillary pulmonary hypertension enrolled in DISCOVER PH-HF

	No-PH (*n* = 7)	Ipc-PH (*n* = 12)	Cpc-PH (*n* = 18)	*P* for trend
Age (years)	79.0 (62.5–86.0)	81.5 (80.0–83.5)	80.5 (76.3–84.8)	0.73
Female sex [*n* (%)]	3 (42.9)	4 (33.3)	8 (44.4)	0.90
BMI (kg/m^2^)	23.8 (22.8–25.3)	22.95 (21.5–24.4)	22.4 (21.0–24.1)	0.42
NYHA class [*n* (%)]				0.42
I	1 (14.3)	1 (8.3)	1 (5.6)	
II	2 (28.6)	8 (66.7)	10 (55.6)	
III	3 (42.9)	3 (25.0)	7 (38.9)	
IV	1 (14.3)	0 (0.0)	0 (0.0)	
Aetiology of mitral regurgitation [*n* (%)]				0.42
Primary	4 (57.1)	7 (58.3)	2 (11.1)	
Functional	3 (42.9)	4 (33.3)	11 (61.1)	
Ischemic	0	1 (8.3)	5 (27.8)	
LVEF (%)	45.0 (27.5–55.0)	51.5 (38.8–60.0)	50.0 (31.3–55.0)	0.64
Heart failure hospitalization within 6 months [*n* (%)]	3 (42.9)	2 (16.7)	9 (50.0)	0.21
Atrial fibrillation [*n* (%)]	3 (42.9)	6 (50.0)	10 (55.6)	0.91
Chronic pulmonary disease [*n* (%)]	1 (14.3)	1 (8.3)	2 (11.1)	1
Hb (g/dl)	14.3 (12.9–14.8)	12.0 (11.43–12.7)	12.4 (10.7–13.6)	0.04
Creatinine (mg/dl)	1.3 (1.2–1.5)	1.3 (1.1–1.8)	1.4 (0.9–2.2)	0.98
eGFR (ml/min/1.73 m^2^)	48.2 (42.1–52.4)	42.8 (33.9–53.2)	45.8 (26.1–65.5)	0.93
NT-proBNP (pg/ml)	1176 (651–3368)	1195 (563–1909)	5532 (2010–8153)	0.006
Beta-blocker [*n* (%)]	7 (100)	9 (75.0)	16 (88.9)	0.38
ACEi [*n* (%)]	0 (0)	1 (8.3)	1 (5.6)	1
ARB [*n* (%)]	1 (14.3)	3 (25)	4 (22.2)	1
Sacubitril/valsartan [*n* (%)]	4 (57.1)	3 (25.0)	3 (16.7)	0.14
SGLT2i [*n* (%)]	6 (85.7)	6 (50.0)	12 (66.7)	0.32
MRA [*n* (%)]	7 (100)	8 (66.7)	16 (88.9)	0.20
Loop diuretic (%)	5 (71.0)	9 (75.0)	18 (100)	0.03

Cpc-PH, combined postcapillary and precapillary pulmonary hypertension; eGFR, estimated glomerular filtration rate; Ipc-PH, isolated postcapillary pulmonary hypertension; LVEF, left ventricular ejection fraction; MRA, mineralocorticoid receptor antagonist; NYHA, New York Heart Association; PH, pulmonary hypertension.

**Table 2:** Pulmonary hemodynamics at the baseline right heart catheterization in the patients without pulmonary hypertension, and with combined postcapillary and precapillary pulmonary hypertension enrolled in DISCOVER PH-HF

	No-PH (*n* = 7)	Ipc-PH (*n* = 12)	Cpc-PH (*n* = 18)	*P* for trend
SBP (mmHg)	130 ± 21	137 ± 35	133 ± 24	0.90
DBP (mmHg)	70 ± 6	70 ± 12	71 ± 8	0.93
HR (bpm)	63 (51–74)	70 (65–73)	75 (69–78)	0.18
mPAP (mmHg)	16.0 (14.5–18.0)	25.0 (23.0–27.0)	30.5 (26.5–34.0)	<0.001
SpO_2_ with wedged catheter (%)	94.9 (94.2–97.4)	94.5 (93.7–96.9)	98.1 (91.4–99.3)	0.72
PAWP (mmHg)	10.1 ± 3.2	18.5 ± 3.9	18.7 ± 5.7	<0.001
CI (l/min/m^2^)	2.4 (2.1–2.8)	2.8 (2.3–3.4)	2.1 (1.8–2.4)	0.02^*^
PVR (WU)	1.4 (1.1–1.7)	1.6 (1.4–1.9)	2.7 (2.4–5.6)	<0.001
RAP (mmHg)	4 (3.5–5.0)	7.5 (5.8–9.0)	8.5 (5.3–10.0)	0.07
PAPI	4.3 (3.5–5.0)	2.8 (2.2–4.9)	2.8 (1.9–4.3)	0.33

Cpc-PH, combined postcapillary and precapillary pulmonary hypertension; HR, heart rate; Ipc-PH, isolated postcapillary pulmonary hypertension; mPAP, mean pulmonary arterial pressure; PAPI, pulmonary artery pulsatility index; PAWP, pulmonary artery wedge pressure; PVR, pulmonary vascular resistance; RAP, right atrial pressure.

* *P* = 0.015 for Cpc-PH vs. Ipc-PH at Dunn post hoc test.

The total time from blood draw during RHC to blood derivative storage at −80 °C, was always less than 1.5 h. A total of 148 PCV and 148 peripheral plasma aliquots, 111 PCV and 111 peripheral serum aliquots, and 37 PBMC pellets were collected. Together, these samples constitute a comprehensive biobank linked to invasive pulmonary hemodynamics.

MR-TEER was successful in all treated patients. The MitraClip and Pascal systems were similarly used irrespective of the hemodynamic features [no-PH: MitraClip in 5 (71.4%) and Pascal in 2 (28.6%); Ipc-PH: 5 (41.7%) and 7 (58.3%); Cpc-PH: 11(61.1%) and 7 (38.9%); *P* for trend 0.46].

At the time of this report, 26 patients would have reached the timepoint of the follow-up RHC. One died within 3 months from enrolment, and another two needed unplanned outpatient visits for decompensated heart failure between 3 and 6 months from enrolment. Four (16%) out of the 25 subjects alive at 6 months accepted to repeat RHC: 3 had Ipc-PH and 1 had Cpc-PH at the baseline RHC.

## Discussion

Despite its high prevalence and prognostic relevance, the biological mechanisms underlying PH-HF remain incompletely defined, particularly across hemodynamically defined subtypes.^4,7^

In animal models of pulmonary hypertension secondary to left heart disease and in autopsies of individuals who died from heart failure with superimposed PH, wall thickening and other histological alterations were detected in both pulmonary arterioles and PCV, suggesting that PH-HF is underlaid with the remodeling of all types of small pulmonary vessels.^8–10^ A preclinical study, in which pulmonary hypertension was induced in piglets by pulmonary vein banding, highlighted 287 differentially expressed proteins between pulmonary hypertension and control veins. A lower number of differentially expressed proteins was found when pulmonary hypertension and control arteries were compared, supporting the hypothesis that vascular cell biology is more modulated in veins than in arteries in PH-HF.^10^ A major role of PCV remodeling can be also inferred by physiology studies in humans. Despite reaching the same PAWP during exercise, subjects with Cpc-PH suffer from more severe lung congestion than those with Ipc-PH, and this finding was attributed to venous involvement with greater increases in capillary pressure in the former than in the latter.^11^ Severe reduction in the diffusing capacity of carbon monoxide can be seen in patients with heart failure and PH, even when they are compensated and, thus, it is unlikely that lung congestion impairs carbon monoxide exchange at the capillary–alveolar membrane. This abnormality can be related to a vasculopathy affecting PCV.^12^

PCV blood, as obtained by RHC, and – for comparison – peripheral venous blood represent valuable sources for examining the levels of proteins and other macromolecules across the stages of human PH-HF, and point out differences between noPH and PH-HF, as well as between Ipc-PH and Cpc-PH.

Indeed, omics of blood samples collected during RHC are at the forefront of translational research in PH. This strategy is one of the work packages of the USA National Institute of Health/National Heart, Lung, and Blood Institute-promoted initiative, Redefining Pulmonary Hypertension through Pulmonary Vascular Disease Phenomics (PVDOMICS), covering all types of PH.^13^ Another recent study evaluated proteome gradients between pulmonary precapillary and postcapillary blood, drawn through an unwedged and wedged Swan-Ganz catheter, respectively, in patients with heart failure and in a limited number of controls.^14^

Medium-throughput proteomics of peripheral venous blood with proximity extension assay has already been carried out in patients with heart failure and MR.^15,16^ In those undergoing TEER, changes in some proteins were related to postprocedural systolic PAP increase.^16^ However, the pulmonary circulation was not directly explored, nor were invasive hemodynamic measurements taken.

DISCOVER PH-HF continues the cutting-edge line of research into PH-HF based on the systematic analyses of RHC-derived blood specimens, and integrates prior research on biomarkers in patients with MR.^17^

Part of the biobank established by DISCOVER PH-HF will be used to perform high-throughput proteomics with the Olink technology. Liquid chromatography–tandem mass spectroscopy of blood is unreliable because of the predominant presence of albumin and other large proteins, and the risk of technical failure is unacceptable in investigations in which samples are invasively obtained such as this one. The proximity extension assay overcomes this issue and still enables the assay of thousands of proteins. This methodology has already and successfully been utilized with peripheral venous blood samples from heart failure patients.^15,16,18^ To our knowledge, DISCOVER PH-HF represents the first systematic application of proximity extension assay-based proteomics to PCV blood obtained during RHC in humans.

After running the proteomic analysis, there will still be substantial amounts of plasma, serum, and PBMC pellets to address additional research questions, by evaluating the presence and/or levels of not only specific proteins but also other macromolecules. Medications and conditions possibly influencing the pulmonary vasculature were carefully excluded and the biological material was handled by following the same protocol at the three study centers, in order to minimize preanalytical variability.

Biological assessments will be complemented by a thorough clinical description of the recruited patients. The DISCOVER PH-HF cohorts replicate typical subgroups of patients with severe mitral regurgitation and heart failure. It is noteworthy that those patients with normal mPAP at the baseline RHC actually had severe MR. The most plausible explanation for their hemodynamic phenotype at the time of enrolment is that effective heart failure therapy had abated a previously increased PAP. In other words, they can be viewed as the mildest extreme of the PH-HF spectrum, which is therefore fully covered by DISCOVER PH-HF.

Although originally planned, the second RHC has minimally been done so far due to patient withdrawal. The analysis of blood samples at baseline and after the treatment of mitral regurgitation, which is anticipated to diminish PAP, would strengthen the potential of DISCOVER PH-HF. Nevertheless, it is not surprising that few subjects have been willing to undergo the follow-up RHC, as it is not part of standard practice. In fact, this limited uptake underscores the challenges of performing invasive studies in real-world heart failure patients.

By giving pathophysiology insights, the biomarkers detected in DISCOVER PH-HF may lay the foundations for the development of novel therapies for PH-HF. This is remarkable given the current lack of specific treatments for PH-HF.

Heart-failure drugs generally reduce PH only to a certain extent.^19^ In 1581 subjects with baseline PVR of at least 3 WU included in the Interagency Registry for Mechanically Assisted Circulatory Support registry, PVR decreased rapidly by around 1.5 WU per month over the first 3 months after the implantation of a left ventricular assist device. Yet, up to 25% of patients had PVR persistently for at least 3 WU 36 months postimplantation.^20^ Clinical trials testing pulmonary vasodilators, which target the pathways underlying arteriolar vasoconstriction and remodeling in pulmonary arterial hypertension, have yielded conflicting results in PH-HF, without clear signals of efficacy, and even safety concerns.^4^ A better knowledge of pulmonary vasculopathy is probably needed to guide therapeutic breakthroughs in PH-HF.

We acknowledge that DISCOVER PH-HF has shortcomings, especially the small sample size and the low rate of longitudinal collection of PCV and peripheral venous blood samples. As an introduction to the study, this article is also limited by the lack of biomarker results.

We decided to exclude patients who had PVR above 2 WU despite normal mPAP, as this hemodynamic pattern is in contrast with the definition of PH set by the guidelines. Nonetheless, it may have biological meaning, which should be addressed by future investigations.

## Conclusion

In conclusion, DISCOVER PH-HF has produced a biobank of PCV and peripheral venous blood samples and PBMCs, linked to clinical and invasive hemodynamic phenotyping. By integrating RHC-derived biological data with clinical and hemodynamic data, this platform will enable the large-scale exploration of biomarkers across the spectrum of PH-HF and may help bridge the gap between pulmonary vascular pathobiology and current hemodynamic classifications.

## Acknowledgements

The authors acknowledge the excellent work done by the personnel of the grant offices of their institutions throughout the study.

Funding: This work was supported by the Italian Ministry of University and Research (PRIN 2022 PNRR funded by the European Union – Next Generation EU; project P20225BYWX, CUP D53D23020960001; PI Italo Porto).

### Conflicts of interest

G.M.D.F. received speaker and/or advisory board fees from Amgen, Daichii, Merck, Novartis and UCB. M.A. received speaker fees from Abbott, Edwards, Lifesciences and Medtronic. P.A. received speaker and/or advisory board fees from Bayer, Daiichi Sankyo, MSD, Janssen, and Gossamer Bio. I.P. received speaker, advisory board, and consultancy fees from SysMedical, Medtronic, Abbott, Edwards, Abiomed, Terumo, Philips, Sanofi, Amgen, Daiichi Sankyo, Astra Zeneca, Bayer, and Pfizer, and research grants from Chiesi, Bayer, Medtronic, and Abbott. The other authors have no conflicts of interest to disclose.
